# Forward-Time Simulations of Human Populations with Complex Diseases

**DOI:** 10.1371/journal.pgen.0030047

**Published:** 2007-03-23

**Authors:** Bo Peng, Christopher I Amos, Marek Kimmel

**Affiliations:** 1 Department of Epidemiology, The University of Texas M. D. Anderson Cancer Center, Houston, Texas, United States of America; 2 Department of Statistics, Rice University, Texas, United States of America; 3 Institute of Automation, Silesian Technical University, Gliwice, Poland; University of Alabama at Birmingham, United States of America

## Abstract

Due to the increasing power of personal computers, as well as the availability of flexible forward-time simulation programs like simuPOP, it is now possible to simulate the evolution of complex human diseases using a forward-time approach. This approach is potentially more powerful than the coalescent approach since it allows simulations of more than one disease susceptibility locus using almost arbitrary genetic and demographic models. However, the application of such simulations has been deterred by the lack of a suitable simulation framework. For example, it is not clear when and how to introduce disease mutants—especially those under purifying selection—to an evolving population, and how to control the disease allele frequencies at the last generation. In this paper, we introduce a forward-time simulation framework that allows us to generate large multi-generation populations with complex diseases caused by unlinked disease susceptibility loci, according to specified demographic and evolutionary properties. Unrelated individuals, small or large pedigrees can be drawn from the resulting population and provide samples for a wide range of study designs and ascertainment methods. We demonstrate our simulation framework using three examples that map genes associated with affection status, a quantitative trait, and the age of onset of a hypothetical cancer, respectively. Nonadditive fitness models, population structure, and gene–gene interactions are simulated. Case-control, sibpair, and large pedigree samples are drawn from the simulated populations and are examined by a variety of gene-mapping methods.

## Author Summary

Complex diseases such as hypertension and diabetes are usually caused by multiple disease-susceptibility genes, environment factors, and interactions between them. Simulating populations or samples with complex diseases is an effective approach to study the likely genetic architecture of these diseases and to develop more effective gene-mapping methods. Compared to traditional backward-time (coalescent) methods, population-based, forward-time simulations are more suitable for this task because they can simulate almost arbitrary demographic and genetic features. Forward-time simulations also allow the researcher to perform head-to-head comparisons among gene-mapping methods based on different study designs and ascertainment methods. Unfortunately, evolving a population generation by generation is a random process, so the fates of disease alleles are unpredictable and there is no effective way to control the disease allele frequency at the present generation. In this paper, the authors propose a simulation method that avoids these problems and makes forward-time population simulation a practical solution for the simulation of complex diseases.

## Introduction

Because of the high cost of data collection, inaccessibility of ancestral information, and complexity of real-world genetic effects, various kinds of computer simulations have been used to evaluate new gene-mapping methods, test hypotheses, and study the evolution of genetic features [1–3]. In addition, genetic epidemiologists routinely use computer-simulated datasets with known disease susceptibility genes and affection status or trait values to evaluate and compare statistical genetic methods. One source of such datasets is the Genetic Analysis Workshop (http://www.gaworkshop.org).

Two main approaches have been used for such simulations, backward-time (coalescent) [4] and forward-time. Backward-time simulations are sample based. Given a sample of unknown genotype, one identifies the common ancestors of individuals and coalesces them according to a stochastic process characterized by evolutionary properties such as mutation, recombination, and migration. After the most recent common ancestor (MRCA) of all individuals is found, the process runs forward in time and assigns genetic information to individuals on the coalescent tree. This method is efficient because it only concerns individuals related to the final sample. It is also flexible in that it can simulate recombination (through ancestral recombination graphs [5,6]) and many mutation and migration models. Despite difficulties in incorporating natural selection into the coalescent theory, recent advances have allowed simulations of selection under a coalescent framework [7–10].

Although coalescent-based methods can simulate many neutral and non-neutral processes with simple selection models, they cannot yet simulate realistic samples of complex human diseases. The greatest problem is that coalescent-based methods only simulate haplotypes (haploid sequences), so they cannot handle diploid-specific effects such as nonadditive selection or penetrance models. Second, if we exclude studies that use neutral markers as disease susceptibility loci (DSL), currently available coalescent methods can only handle one DSL under constant selection pressure [10]. This limits their ability to model the evolution of complex human diseases, which, although largely unexplored, is likely influenced by varying selection pressure on more than one DSL [11]. Finally, the coalescent simulations are based on a series of approximations and equilibrium assumptions and are supposed to work only for certain parameter ranges [12], such as low recombination and mutation rates.

Forward-time simulations are simpler as an idea. A forward-time simulation usually starts from an initial population and follows its evolution generation by generation, subject to a certain number of genetic or demographic changes. Population properties can be observed at each generation, and samples are drawn from the last several generations. Because there are no theoretical constraints, this approach can theoretically simulate arbitrarily complex evolutionary scenarios as well as complex selection and penetrance models. Although this method is less efficient than the coalescent approach, the continuing increase of the power of computers, and the availability of efficient computer programs such as easyPOP [13] and simuPOP [14], have made realistic forward-time simulations possible in genetics studies [3,15–17].

The effective use of forward-time simulations in sample generation is, however, deterred by the lack of a suitable simulation framework. Although many simulations have been run for different purposes, the simulation scenarios differ greatly [3,18]. Currently, there are no definite solutions to the following problems: (i) *Simulation length and initialization.* Unlike the coalescent approach, which starts from a single individual (MRCA), forward-time simulations usually start from an initial population of moderate size. How to initialize this population is a surprisingly difficult question. (ii) *Introduction of disease.* In the coalescent approach, the age of the mutant is random, because the age of the MRCA of all affected individuals is random. This is difficult to achieve using a forward-time approach. A more serious problem is that newly introduced disease mutants, especially those under purifying selection, tend to be lost quickly, and simulations may have to be repeatedly restarted. (iii) *Control of disease allele frequency.* The forward-time approach is directly affected by genetic drift, making it difficult to control the allele frequency at the ending generation, which makes a fair comparison of simulated samples difficult.

In this paper, we will introduce a forward-time simulation framework that allows us to generate large multi-generation populations with complex diseases according to specified demographic and evolutionary properties and allele frequencies at the present generation. Arbitrary study designs, ascertainment methods, and gene-mapping methods can be applied to the simulated populations.

We demonstrate the use of these populations using three examples. In the first example, we apply the Linkage test, transmission disequilibrium test (TDT) [19], and χ^2^-association test to affected sibpair and case-control samples drawn from simulated populations with different levels of population structure. Additive fitness and penetrance models are used. In this example, gene-mapping methods based on different study designs are compared directly, which can rarely be done using other simulation methods. The second example involves a quantitative trait that is affected by three nonadditive DSL and an environmental factor. Variance components [20] and variance regression [21] methods are applied to sibpair families and large pedigrees. The last example simulates the evolution of an inheritary cancer with individual affection status and age of onset affected by three interacting DSL. Logistic regression and Cox proportional hazards models [22] are used to analyze samples from two case-control designs.

## Methods

We define a population as a collection of diploid individuals with the same genotype structure, represented by the number of chromosomes, number of markers, their type (e.g., SNP or microsatellite), and positions on the chromosomes. During evolution, individuals are chosen randomly, with probabilities that are proportional to their relative fitness values, to mate and produce offspring that populate the next generation. Mutation, migration, recombination, and demographic changes shape the genetic features of a population. When subpopulations are present, mating occurs within subpopulations, and exchange of genetic information between subpopulations can only occur through migration.

We assume that a genetic disease is caused by mutations at one or several diallelic DSL. They are located on different chromosomes and are thus unlinked. (Our algorithm cannot yet handle linked DSL.) These loci only have wild-type alleles until one mutant (disease allele) is introduced at each locus. These disease mutants spread in the population during evolution, subject to genetic drift and positive or negative selection pressure, reaching certain allele frequencies at the present generation. We assume that we know the exact demographic history (the size of population and number and sizes of subpopulations at each generation) and the selection pressure for all genotypes. The selection pressure may vary during evolution.

To simulate the evolution of this disease, we propose the following simulation framework: (i) Given current disease allele frequencies and selection and demographic models, simulate trajectories of allele frequencies at each DSL using a backward approach. (ii) Create an initial population and initialize individuals randomly with several initial haplotypes. Burn in the population subject to mutation (non-DSL markers only) and recombination, which will be present during the whole evolutionary process. (iii) Introduce the disease alleles to the population by point-mutating disease loci of different individuals. The generations when mutants are introduced are determined by the length of allele frequency trajectories. (iv) Evolve the population according to the simulated allele frequency trajectories and predetermined demographic features. A typical simulation scenario will split the population into subpopulations, and evolve them independently for a number of generations. This can be followed by a mixing stage, during which migration is allowed. This process allows population differentiation to be first built up and then attenuated. (v) Save the last several generations as the resulting population. To facilitate subsequent analyses, random mating does not have to be used during this sample preparation stage. For example, the number of offspring per mating event can be increased to generate more sibpairs.

The advantage of this approach is that we control the disease allele frequencies during evolution while allowing random introduction of disease mutants. The following subsections explain details of the method.

### 

#### Simulating the trajectory of disease allele frequency.

The idea of trajectory simulation has been used by others [10,23] in the context of coalescent simulations. For example, Wang and Rannala [23] used an additive selection model and a forward approach with a normal approximation to the binomial selection process. This method can handle one DSL and arbitrary demographic models. Coop and Griffiths [10] used diffusion approximation and a backward approach to simulate the trajectory of the allele frequency of a single locus in a population with a constant size. Our method extends these methods, and the method described in Slatkin [24].

We assume that the population size at generation *t* is *N_t_*. The locus discussed is diallelic with wildtype allele *A* and disease allele *a*. Relative fitnesses of genotypes *AA, Aa,* and *aa* are 1, 1 + *s*_1_, and 1 + *s*_2_, respectively. *s*_1_ and *s*_2_ can assume any value greater than −1. Allele *a* is called advantageous if *s_i_* > 0, and deleterious if *s_i_* < 0 (*i =* 1, 2). *s*_1_ and *s*_2_ can take different signs, as in the case of balanced selection.

Suppose that disease allele *a* is introduced to a population at generation 1 and spreads according to a Wright-Fisher model with varying population size and a selection model described above. At generation *T*, the population is surveyed and *i* copies of allele *a* are found. We are interested in simulating the trajectory *H =* {*i*_0_ = 0, *i*_1_ = 1, …, *i_T_ = i*}, where *i_t_* is the number of copies of allele *a* at generation *t*. The length *T* of the trajectory is the age of the mutant.

The dynamics of allele frequency *x_t_* can be modeled as follows: Assume that at generation *t −* 1 there are *i_t_*_− 1_ copies of allele *a*. Population allele frequency is equal to 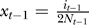 . Assume that the next generation is formed from an infinite-sized gene pool. The expected frequency of allele *a* at generation *t* is expressed by

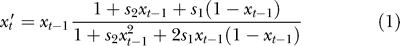
[24]. Therefore, the probability that there are *i_t_* copies of allele *a* at generation *t*, given population size *N_t_*, equals

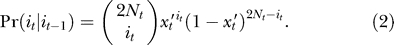



We use 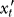 to denote expected allele frequency as opposed to the real allele frequency 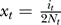 . The probability of trajectory *H =* (*i*_0_, *i*_1_, …, *i_T_*) equals

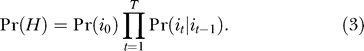



#### Forward- and backward-time simulations.

Formulas 1 and 2 provide a way to simulate allele frequency trajectories in a forward-time manner. One may start from a single disease mutant and simulate allele frequencies at each generation until generation *T*. The resulting trajectory will be accepted if *x_T_* is within the designed range, or rejected otherwise.

This algorithm works in principle and is used by programs such as GeneArtisan [23]. However, it suffers from several major problems: (i) If *T* is large, the disease allele is under strong purifying selection, or the acceptance region is too narrow, the acceptance probability of a trajectory will be small. Obtaining one valid trajectory may require millions of attempts. (ii) This method assumes a fixed *T*, but *T* is usually random. Unbiased samples of the trajectories can only be simulated if *T* is chosen randomly from its distribution, which is usually unknown. If an inappropriate *T* is chosen, the simulated trajectories will be biased.

An alternative to the forward-time algorithm is a backward approach, which was first explored by Slatkin [24] in a monogenic disease setting. Using this approach, a trajectory can be generated by a model that assumes *i* copies of allele *a* at *t = T* and proceeds backward in time until the allele is lost. The generation at which the allele is lost becomes generation 0, if there is exactly one copy of allele *a* at generation 1. This approach avoids the problems of the forward-time approach. Note that the quality of a backward-time method should be evaluated from an importance-sampling perspective [25], because a backward algorithm may not generate the same trajectories with the same probabilities as the forward approach [24].

#### Reversible trajectories.

Equation 2 is a transition probability of a Markov process. If this process is reversible, we can simulate a trajectory starting from *i* alleles, going back in time until all alleles are lost, and obtain Pr(*H =* {*i*_0_ = 0, *i*_1_, …, *i_T_ = i*}), with a probability identical to that based on the forward approach. The reversibility properties of various cases of Wright-Fisher processes, with or without selection and with constant or varying population size, have been studied extensively [24]: (i) The Moran model with selection and mutation is reversible [26]. In the Moran model, the probability distribution of times to loss of an allele is the same as the distribution of allele ages. (ii) The Wright-Fisher model in a population of constant size is *not* reversible. However, because the diffusion limit of the Wright-Fisher model, a continuous-time approximation to the discrete-time Markov model, is the same as that for the Moran model, the Wright-Fisher model is approximately reversible in a constant population [27]. (iii) In the case of constant population size and an additive advantage selection model, Maruyama [28] showed that, in the diffusion limit, the distribution of allele age is invariant to the change of sign of the selection coefficient.

For these reversible processes, we can simulate a trajectory by starting from current allele frequency *i*/2*N* and evolve it randomly using 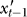 = *x_r_* for case 2 and 
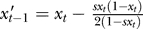
 (*s*_2_ = 2*s*_1_ = *s*) for case 3 until the allele is lost. The sign of selection pressure is reversed for the reversal processes.

#### Nonadditive alleles and variable population size.

For an allele with a nonadditive effect on fitness, the change of sign of the selection coefficient is not equivalent with time reversal. For variable population size, the Markov process does not have a stationary distribution and thus is not reversible in the usual sense. Techniques applicable in such situations have been proposed and tested by Slatkin [24].

The basic idea is to match the forward process as close as possible by reversing Formula 2 with an appropriately inverted Equation 1. This can be achieved by solving the equation

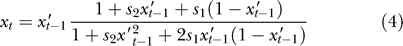
for 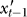 , with *i_t_, x_t_, N_t_*, and *N_t−_*_1_ given. Equation 4 is obtained from Equation 1 by replacing *x_t −_*_1_ (now unknown) by 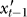 and replacing 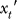 (now known) by its sample value *x_t_*. Given *N_t−_*_1_ and 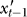 , we can then simulate *i_t−_*_1_ by

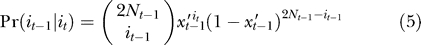



This process is feasible because Equation 4 is a quadratic equation that has a unique solution (between 0 and 1) in all combinations of *s*_1_, *s*_2_, and *x_t_* (proof ignored).

#### Varying selection pressure.

The distribution of *i_t_* (Equations 1 and 2) concerns only the selection coefficient at generation *t* − 1; thus, it also works in cases of varying selection pressure. The backward process can also be adapted to work for varying selection pressure. Given the selection coefficient *s_i,t−_*_1_ at generation *t −* 1 and the allele frequency *x_t_* at generation *t,* the expected allele frequency at generation *t −* 1 can be obtained by solving Equation 4 with *s*_1_ and *s_2_* replaced by *s*_1*,t−*1_ and *s*_2,*t−*1_, respectively. If *s*_1*,t−*1_ and *s*_2*,t−*1_ are not constant (e.g., depending on the allele frequency *x_t−_*_1_), we can replace *s*_1_ and *s*_2_ with 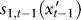 and 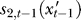 . If there is no easy solution to Equation 4 in the latter case, using *s*_1,*t−*1_(*x_t_*) and *s*_2*,t−*1_(*x_t_*) is usually sufficient because *x_t_* values in successive generations do not differ much, and *s_i_* is usually not sensitive to small allele frequency changes.

**Figure 1:**
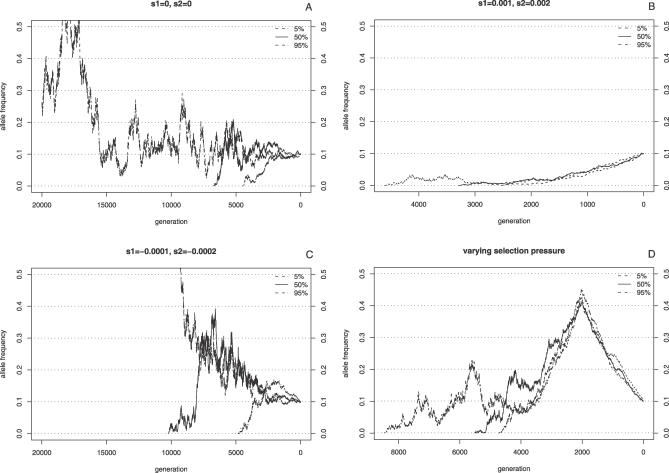
Examples of Simulated Trajectories Trajectories of simulated allele frequency under different selection models. For each selection model, 100 replicates are simulated and three trajectories corresponding to the 5%, 50%, and 95% quantiles of the trajectory length are plotted. The selection models are neutral (left top, *s*_1_ = *s*_2_ = 0), advantageous (right top, *s*_1_ = 0.001 and *s*_2_ = 0.002), deleterious (left bottom, *s*_1_ = −0.001 and *s*_2_ = −0.002) and a mixed-selection model (right bottom) in which the disease allele is advantageous before 2,000 generations ago (*s*_1_ = 0.001 and *s*_2_ = 0.002) and is under purifying selection in the recent 2,000 generations (*s*_1_ = −0.001 and *s*_2_ = −0.002). In all cases, the current allele frequency is 10%. The population size is

Figure 1 displays sample trajectories of neutral, advantageous, deleterious mutants, and mutants under varying selection pressure (Figure 1A, 1B, 1C, and 1D, respectively), using the same exponential growth demographic model. In the last case, alleles were advantageous 2,000 generations back and became deleterious afterward. The three curves in each panel correspond to trajectories with lengths that are the 5%, 50%, and 95% quantiles of the lengths of 100 simulated trajectories. From these figures, it is evident that allele frequencies oscillate less when population size becomes larger. Trajectories under advantageous selection pressure (Figure 1B) are also smoother than are those under neutral or purifying selection (Figure 1A and 1C). This is because many trajectories can naturally reach high allele frequency under advantageous selection, but alleles under purifying selection usually need to reach a higher frequency by chance to compensate for selection and avoid extinction before they reach the present generation.

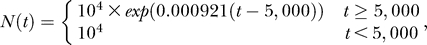
such that *N*(10,000) = 10^6^. Note that one of the trajectories in the left bottom panel is longer than 20,000 generations and its allele frequency is more than 0.5 before generation 10,000.

#### Trajectories of DSL of polygenic diseases.

If the multi-locus selection of a polygenic disease is modeled by additive or multiplicative multi-locus models, it is sufficient to simulate the trajectory of each DSL independently because the evolution of the DSL will be largely independent [17].

The problem is more complicated when disease loci interact with each other. In these cases, the fitness value of an individual at a DSL (*X = A*) is influenced by the genotype at other DSL (*Y =* (*B, C, …*)). If DSL are unlinked, we can estimate the proportions of all genotypes as the product of single-locus genotype frequencies (*P*(*XY*) = *P*(*A*)*P*(*B*)*P*(*C*) …). The population average of the fitness at a DSL is then the weighted average of the fitnesses of all genotypes. For example,

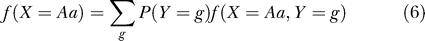
where *f*(·) is the fitness value of genotypes. The trajectory of locus *X* can then be simulated using the method for frequency-dependent selection. Our simulation method cannot yet handle cases with linked loci because *P*(*Y*) in Equation 6 cannot be easily calculated when *Y* is composed of more than one DSL.

#### Population structure and migration.

We aim to control the total disease allele frequencies of DSL at the present generation. With the presence of subpopulation structure, it is necessary to divide the total expected number of disease alleles among subpopulations.

If disease allele frequencies in each subpopulation at the present generation are not specified, our implementation allows specification of two ways, even or uneven, of distributing disease alleles among subpopulations. In the even case, a multinomial distribution is used to distribute disease alleles among subpopulations, with probabilities proportional to subpopulation sizes. This distribution models the random assignment of disease alleles to subpopulations and results in roughly equal numbers of disease alleles among equal-sized subpopulations. In the uneven case, assuming there are *m* equal-sized subpopulations, *m −* 1 random numbers between 0 and 1 are placed on (0, 1), and the interval lengths (*l_i_*) between adjacent points become the weights at which disease alleles are distributed. This process models a Poisson process with constraint 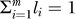 so the differences between *l_i_* are larger than the multinomial case.

In the case of no migration, we simulate trajectories in each subpopulation independently. Subpopulations can have different demographic models and the subpopulation specific trajectories are simulated in the usual way, with the restriction that all disease mutants are introduced before a population split. At the generation when the population splits, simulated trajectories from each subpopulation are combined and the process is continued in the single before-split population.

Migration can be incorporated into this simulation framework. Adjustment of disease allele frequencies is needed at each generation according to the specified migration model. However, this complicated process can be ignored if a symmetric migration model is used on equal-sized subpopulations with an even distribution of disease alleles because migration will have little impact on the allele frequencies in the subpopulations.

#### Demographic models and initialization.

*Demographic models.* Real human populations have different demographic histories. Some populations, such as the Scandinavian Saami isolate, have an approximately constant population size. Others have experienced recent bottleneck, are composed of several small tribes with almost no migration, or have undergone a rapid population expansion [29]. To study the evolution of human diseases in different populations, different demographic models are needed.

An example of a commonly used model [1] is an exponential growth model in which a population starts to expand about 80,000 y ago from a founder population of size *N*_0_ = 10^4^ to its current population of size *N*_1_ = 10^6^. If we assume 20 y per generation, 80,000 y correspond to 4,000 generations. The population size function, in the unit of generation, is then

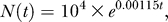
where rate *r* is calculated from *N*(4000) = *N*_0_ × *e* ^4000*r*^.

*N*_1_ in this model is not the effective population size of the final population because of population expansion [30]. It cannot match the census population size of the present human population (6 × 10^9^) or even regional populations such as that of the United States. However, because the effective population sizes of real human populations (at the order of 10^4^) are far below their census sizes because of population structure and nonrandom mating, *N*_1_ = 10^6^ should be able to mimic the genetic diversity of regional populations. For example, it has been shown that *N*_1_ = 10^6^ suffices in the study of the evolution of allelic spectra of human diseases [17].

*Initialization.* The genotype of the population when the disease mutant is introduced has a strong impact on the final sample, if not on the disease locus itself. For example, if linkage disequilibrium (LD) between adjacent loci is strong when a disease mutant is introduced, it will tend to dissipate LD between DSL and their surrounding markers and affect the mapping of the DSL using LD-based methods.

The coalescent approach does not have this problem. Because the MRCA is known, all recombination events on the coalescent tree are explicitly specified and the level of LD is determined by the age of MRCA and the recombination rate. The LD level at the generation when the disease mutant is introduced is determined by time elapsed from the MRCA. Because the age of MRCA estimated from the coalescent theory is often in the order of 4*N*, which is roughly the length of human history (1,000,000 y) for a population of effective population size 10,000 [31], simulating such a long period of time seems unacceptable.

We use a small founder population and a burn-in process to control the properties of the initial population. At the beginning of a simulation, a small founder population is created and is initialized with several haplotypes. The population, with initial complete LD, will then evolve for a few thousand generations to allow for the degeneration of LD between markers.

The length of burn-in is determined by the nature of a simulation. In one example, the recombination rate between adjacent markers is 0.0005, which corresponds to roughly 50,000 base pairs. Because the LD between two markers at this distance is usually moderate on human chromosomes [32], we burn-in the initial population for 4,000 generations before another 6,000 generations of evolution time. The level of LD in the final generation is then approximately (1 − 0.0005)^10000^ = 0.0067. This corresponds to *r* ^2^ = 0.0055 for two loci with a minor allele frequency of 0.1, a level that is consistent with empirical and theoretic estimates on most regions of human chromosomes, in the absence of a substantial population structure [32]. The burn-in length will be more important for the simulations of denser markers, when the LD is expected between adjacent markers and its level is controlled by the length of evolution.

#### Random mating with controlled disease allele frequency.

With simulated allele frequency trajectories of the DSL, it is necessary to develop a method to perform random mating while controlling the disease allele frequency during evolution. The rejection sampling algorithm, in which the next generation is regenerated if its allele frequency does not match the simulated one, can be used in principle. However, this algorithm is not efficient for practical use, especially when more than one DSL is involved.

Controlled random mating has been used in the framework of coalescence in the case of haploid populations [33,34]. The algorithm separates generation *t −* 1 and *t* into case and control groups and generates offspring of the case and control groups at generation *t* from their counterparts at generation *t −* 1.

The above works for a haploid population with one DSL because of the independent segregation of wild-type and disease alleles. However, it does not work for a diploid population in which the wild-type and disease alleles cosegregate as heterozygotes. For a diploid population, we propose the use of an approximate algorithm. This algorithm splits the random mating process into two stages: (i) A reject-sampling method is applied so that only individuals with disease alleles are accepted until we obtain enough disease alleles to fit the simulated frequency trajectory, and (ii) only individuals with no disease allele are accepted; they fill the rest of the offspring generation.

Cosegregation of multiple loci because of selection against multiple DSL complicates the problem even more. It is difficult, and sometimes impossible, to satisfy allele frequency requirements at all DSL. Rather than one of several more complicated algorithms, we choose a simple extension to the diploid algorithm. During the first stage of this algorithm, we accept individuals that have any of the needed disease alleles until the frequency requirements at all DSL are met. The second stage proceeds as usual. An obvious problem with this algorithm is that at the end of the first stage, disease alleles at some DSL are accepted even if the allele frequency requirements at these DSL have been met. This will result in, on the average, more disease alleles at all DSL. The impact of this problem is generally negligible and is discussed in the Discussion section (see examples in Table 1).

**Table 1:**
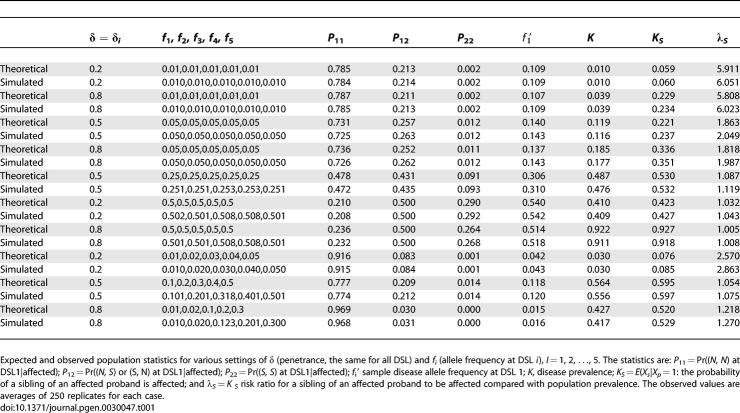
Theoretical versus Simulated Population Statistics

In forward-time simulations, one mating event can produce more than one offspring. Because the relationship between offspring of the same family is important for gene-mapping methods, family structure is preserved whenever possible. In the implementation of all the algorithms described above, acceptances and rejections are family based. Namely, the whole family is accepted or rejected, depending on its contribution to the number of disease alleles.

**Figure 2:**
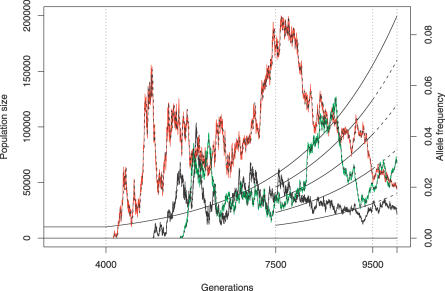
Illustration of the Evolutionary Scenario Illustration of the evolutionary scenario of a simulation with three DSL. Demographic model (left axis): The population starts at size 10,000 and begins to grow exponentially at generation 4,000. The population is split into five equal-sized subpopulations at generation 7,500 (with subpopulations separated with solid lines) and reaches size 2 × 10^5^ at generation 10,000. Migration is allowed from generation 9,500 to 10,000 (with subpopulations separated by dashed lines). Disease allele frequencies (right axis): The DSL are under advantageous selection pressure, with fitnesses of 1, 1.0001, and 1.0002 for genotypes *AA, Aa,* and *aa*, respectively, where *a* is the disease susceptibility allele. The present disease allele frequencies are 0.01, 0.02, and 0.03, respectively. The trajectories simulated backward in time are plotted in solid lines with different colors. The trajectories obtained during forward-time controlled random mating are plotted as dotted lines, which are indistinguishable from the simulated trajectories.

As a summary of all the described steps, Figure 2 plots a typical simulation, in which three DSL contribute to a polygenic disease. Disease mutants are introduced around 5,000 generations into the past, subject to advantageous selection pressure with fitness 1, 1.0001, or 1.0002 for genotypes *AA, Aa,* or *aa,* respectively (where *a* is the disease allele), and reach their present allele frequency 0.01, 0.02, and 0.03. The demographic model involves exponential population growth, population split and mixing.

#### Examples.

Unlike other simulation methods that produce samples of certain formats, our simulation method yields large multigeneration populations with designed disease allele frequencies. Affection status, quantitative traits, and other properties such as age, age of onset, and stage of disease can be attached to individuals according to individual genotype and environmental factors. Multiple samples of different formats can be drawn from these populations. This allows for analyses of the whole population and comparisons between not only samples but also study designs and ascertainment methods.

We demonstrate the use of such simulations using three examples. In all examples, we use a fitness model that is independent of individual affection status or trait values and bypass modeling penetrance in our simulations. This is because our simulations follow the allelic composition of a population, which is the result of mutation, selection, and genetic drift. The affection status or trait value is assumed to reflect the same underlying genotype as fitness, and its impact on the allelic composition of the next generation is represented by fitness. For example, if individuals with genotype *AA, Aa,* or *aa* are affected with a probability of 0, 0.2, or 0.8 and affected individuals have a probability of 0.5 to be removed or not produce offspring, we can replace this two-step penetrance/selection model with an equivalent selection model that works directly on the genotype, namely a selection model that has a fitness of 1, 0.9, or 0.6 for genotypes *AA, Aa,* or *aa,* respectively.

Another commonly used strategy is to increase the number of offspring per mating event during the sample-preparation stage. For example, we change the number of offspring to two in Examples 1 and 2. This is because the probability of getting one full sibship is 1/*N* ^2^ (*N* is population or subpopulation size) using the usual random mating scheme, which makes it impossible to sample pedigrees with multiple offspring in the resulting population. The impact of this change is discussed in the Discussion section.

We use various gene-mapping methods to map DSL in the examples. These methods are chosen for convenience and popularity rather than their suitability for the analyzed datasets. None of the gene-mapping methods used in Example 1 considers the fact that all DSL have undergone positive selection. Because the mapping results are used for demonstration and comparison purposes, the *p*-values are not adjusted for potential multiple testing problems.

*Example 1: The impact of population structure on the power of gene-mapping methods.* In this example, each individual has four chromosomes that each have 20 SNP markers. The markers are spread evenly over all chromosomes, with 1, 2, or 3 DSL placed at the center of the first 1, 2, or 3 chromosomes (between markers 10 and 11). The SNP markers are mutated using a two-allele Jukes-Cantor [35] mutation model. Note that back mutation is allowed and has the same mutation rate as forward mutation. This is different from the infinite-site mutation models frequently assumed in coalescent simulations.

Physical locations of markers are not explicitly specified and are roughly determined by recombination rates between adjacent markers. We use *r =* 0.0005 in this example, which corresponds to 0.05 centiMorgan (around 50,000 base pairs) between adjacent markers and approximately 1 centiMorgan for the whole chromosome (using Haldane's mapping function 
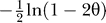
 , where θ = 19 × 0.0005). Recombination is uniform on the chromosomes, and the recombination rate between DSL and its adjacent marker is half of the value between markers because it is halfway between SNP 10 and 11.

An additive fitness model is used. That is, fitness at the DSL with genotype *NN, NS,* or *SS* is 1, 1 + *s*/2, or 1 + *s*, respectively, where *s =* 0.001 is the selection coefficient. The overall fitness value is obtained using a multiplicative model [2,36]. The affection status of each individual is assigned according to a heterogeneity model [36] superimposed on an additive model at each DSL. Namely, the penetrance at a DSL with *NN, NS,* or *SS* is 0, δ/2, or δ, respectively, and the overall penetrance is determined by

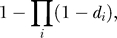
where *d_i_* is the penetrance value at locus *i*. We use δ*_i_* = 0.5 for all DSL.

Our simulations increase the initial population from *N*_0_ = 10^4^ to *N*_1_ = 2 × 10^5^ in 5,000 generations, after 5,000 burn-in generations. Disease mutants are introduced between 3,500 and 4,000 generations ago and reach disease allele frequencies 0.05 at the present generation. Most simulated trajectories fall in this age range, and we exclude trajectories with younger or older mutants to minimize differences between simulated populations.

We simulate three demographic models: no population structure, population structure with even distribution of disease alleles among subpopulations, and population structure with uneven distribution of disease alleles among subpopulations. In the latter two cases, the populations are split into ten subpopulations at 2,000 generations ago. The level of population differentiation is measured by *F_ST_,* calculated using the method introduced by Weir and Cockerham [37].

We draw affected sibpair, as well as case control samples from the present generation. The affected sibpair samples consist of 200 families with two affected offspring and their parents. The case control samples consist of 400 cases and 400 controls. We assume that we cannot observe the DSL directly so the disease loci are removed from the samples.

Two popular family-based gene-mapping methods, TDT [19] and Linkage methods, are applied to affected sibpair samples. The χ^2^ association test is applied to case-control samples. Power is calculated as the proportion of tests with a *p*-value of ≤0.05 at markers 11 (right next to the DSL) and 16 on each chromosome with a DSL; The type-I error is calculated as the proportion of tests with a *p*-value of ≤0.05 at marker 11 on chromosomes without DSL. We use the single-locus TDT method in GeneHunter [38,39] for the TDT analyses and the multi-locus nonparametric method in Merlin [40] for the Linkage analyses. This method invokes a procedure described by Kong and Cox [41] to allow for adjustment for incomplete information.

*Example 2: Mapping a quantitative trait using small or large pedigrees.* Individuals in this example have four chromosomes, with three DSL located at the center of the first three chromosomes. There are 20 SNP markers on each chromosome, with the same mutation model and uniform recombination rate in the first example. The recombination rate is *r* = 0.005, which corresponds to chromosomes of 10 centiMorgan in length.

Different selection models are used at each DSL. The heterozygotes (*Aa*) at the first DSL are advantageous with *s*_1_ = 0.005, whereas its homozygotes (*aa*) are deleterious with *s*_2_ = −0.001. This models a DSL with a heterozygous advantage. The second DSL is advantageous, but only in the form of homozygotes (*s*_1_ = 0 and *s*_2_ = 0.05). The third DSL is peculiar in that its heterozygotes are deleterious and its homozygotes are advantageous (*s*_1_ = −0.001 and *s*_2_ = 0.1). A multiplicative multi-locus fitness model is used. The allele frequencies of all three DSL are 0.20 at the present population. With a demographic model that a population of size *N*_0_ = 10^4^ increased exponentially to *N*_1_ = 5 ×10^5^ in 5,000 generations after 5,000 generations of burning-in, mutants at the three DSL are, on average, 1,600, 3,400, and 4,000 generations old, respectively. Note that it is difficult to simulate DSL under purifying selection with this demographic model and such high present disease allele frequencies.

A quantitative trait is affected by these three DSL, and its value is determined by

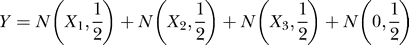
where *X_i_* is the number of mutants (0, 1, or 2) at the *i*th DSL and *N*(*a, b*) is a normal distribution with mean *a* and standard deviation *b*. In this model, all mutants have the same impact on the quantitative trait, regardless of single-locus fitness models.

The last three generations are saved as the final population. We draw small and large pedigrees from the final populations and form samples of size 800. Sibpair samples consist of 200 random sibpairs and their parents. Large pedigree samples consist of three generation pedigrees of at least eight individuals. These pedigrees have two grandparents, parents, spouses, and children. We apply variance components [20] and variance regression [21] methods to these samples. Merlin [40] and Merlin-regress [21] are used for the analyses. We estimate power and type-I error in the same way as in the first example, using *p*-values from one half a χ^2^ distribution [21].

*Example 3: Age of onset of a hypothetical cancer caused by three interacting DSL.* Examples 1 and 2 do not adequately reflect the complexity of the evolution of complex human diseases, which usually involves interaction between DSL. In this example, a hypothetical cancer is caused by three DSL *A, B,* and *C* and an environmental factor. None of the DSL are deleterious on their own, and they can be protective in some cases. Cases with a higher risk of cancer occur when homozygotes or heterozygotes of a disease allele at DSL *A* are accompanied by at least one disease allele at DSL *B* or DSL *C*. However, the wild-type homozygote on DSL *A* confers a selective advantage, without regard to the genotype of DSL *B* and *C*. The fitnesses of all genotypes at these DSL are given in Table 2.

**Table 2:**
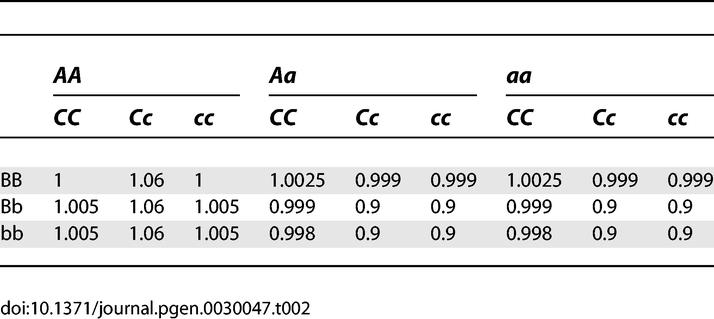
Fitness of Genotypes for Example 3

The genotype structure (chromosomes, markers, and location of DSL) and the demographic model are identical to that of Example 2, but the final population size is larger, with *N*_1_ = 10^6^. The present allele frequencies of the three DSL are 0.3, 0.1, and 0.05 respectively. The average ages of the mutants are 4,700, 4,000, and 800 generations, respectively. Unlike in Examples 1 and 2, we continue to use random mating with one offspring per mating event in the sample preparation stage.

We simulate the age of onset of this cancer for each individual in the present population using a proportional hazards model. The base hazard function is defined as *h*_0_(*t*) = 0.0001*t*, where *t* is age. Corresponding to the selection model, the individual hazard function *h_i_*(*t*) is proportional to the base hazard function

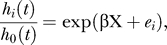
where

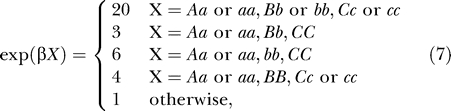
and *e_i_* ∼ *N*(0, 1).We assign a random year of birth to individuals so that the population age distribution is uniform between 0 and 70 y, which roughly corresponds to the age distribution of the population of North America [42]. Individuals with an age of onset older than their age are considered unaffected. We do not model the survival time of affected individuals, and all affected individuals are assumed to be ascertainable, with a known age of onset.

Two kinds of case-control samples are sampled from the population to detect DSL responsible for the disease or the early onset of the disease. The first type of samples use affected individuals as cases and unaffected individuals aged ≥50 y as controls. The second type of samples use affected individuals aged <40 y as cases and affected individuals aged ≥40 y as controls. Logistic regression and Cox proportional hazards models [22], with different interaction terms, are applied to the samples. We use statistical package R to perform the analyses.

#### Electronic resources.

The trajectory simulation algorithm and controlled mating schemes are implemented in simuPOP [14]. A simuPOP script simuComplexDisease.py that implements the simulation scenario is distributed with simuPOP, under the GPL license. The number of markers and population size is only limited by available physical RAM, and execution time increases roughly linearly with an increasing number of markers and population size. A simulation such as that shown in the first example (10^4^ initial population size, 2 × 10^5^ final population size, exponential population growth, 60 markers on three chromosomes, 10^4^ generations) requires approximately 45 min to complete on a workstation with a 2.8G Hz Xeon processor and 2Gb of RAM. We used PC clusters to perform all simulations. Note that a Message Passing Interface version of simuPOP is being developed to take advantage of multi-core and cluster machines, and will allow faster simulations of a large number of markers, as generated by genome-wide association studies.

## Results

### Validation of Simulations

**Figure 3:**
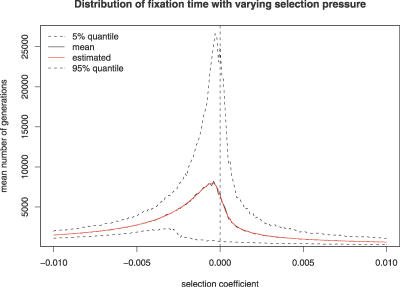
Validation of Trajectory Lengths Mean, 5%, and 95% quantile of the length of trajectories of a mutant under different selection pressure. The mutant starts at allele frequency 0.1, evolves backward in time in a constant population with size *N =* 5,000, and is subjected to constant selection pressure with a selection coefficient *s* of −0.001 to 0.001, until it is lost or fixed. The red smooth curve represents theoretical estimates of the mean number of generations before this mutant is lost or fixed. Note that the simulated trajectories that are fixed or have more than one mutant at generation 1 are also accepted, in accordance with the theoretical estimates.

Theoretical estimates for the distribution of the absorption time of a mutant are available for some simple cases [43]. Because the trajectories of mutants that are neutral or under additive selection pressure in a constant population are reversible, we simulate 1,000 trajectories of such processes backward in time, subject to varying selection pressure (−0.01 ≤ *s* ≤ 0.01), and compare the mean trajectory length with the theoretical estimates of the fixation time of the forward-time processes. The length of theoretical and simulated trajectories match well (Figure 3). Note that when starting allele frequency <0.5, deleterious mutants have longer trajectories than neutral mutants because such mutants are advantageous in a forward-time process and are more likely to be fixed than extinct.

We validate the controlled random mating scheme of our simulations using many different genetic and demographic models. The controlled random mating processes follow simulated trajectories of disease allele frequencies well and reach designed disease allele frequencies. Figure 2 shows one such simulation, where pre-simulated trajectories using a backward approach and recorded frequency trajectories during forward-time simulation are indistinguishable, indicating perfect matches between trajectories.

A nonrandom selection of families may cause disproportionate representation of families with certain configurations of disease alleles. For example, families with multiple disease alleles may be favored because of their higher probabilities of selection. Also, higher allele frequencies at the DSL tend to yield higher than expected allele frequencies because of an increased level of cosegregation at the end of the first stage of controlled random mating. To determine the impact of these potential problems on the statistics of the resulting population, such as sibling recurrence risk ratio, the theoretical and simulated statistics for 2,500 simulated populations (250 replicate for each case) are evaluated and listed in Table 1. The controlled random mating algorithm works, even in cases with high disease allele frequencies. For example, a less than 1% deviation of allele frequencies is observed for the cases with five DSL with 50% disease alleles. Note that many of the theoretical estimates in Table 1 are extensions of the two-DSL cases in [44]. Their mathematical derivations are presented in the supplementary data file (Dataset S1).

### Example 1: The Impact of Population Structure on the Power of Gene-Mapping Methods

The selection and penetrance models we use place equal weight on all DSL. Because most affected individuals have only one mutant (Table 1), these multi-locus models effectively diversify the causal gene of a disease compared with the corresponding single locus model.

We simulate 1,000 replicates for each of the nine cases (1, 2, and 3 DSL, in 3 demographic models). The average *F_ST_* for populations with population structure is 0.974, with little difference between cases with even and uneven distribution of disease alleles at the present generation. The powers and type-I errors of the three gene-mapping methods are listed in Table 3.

**Table 3:**
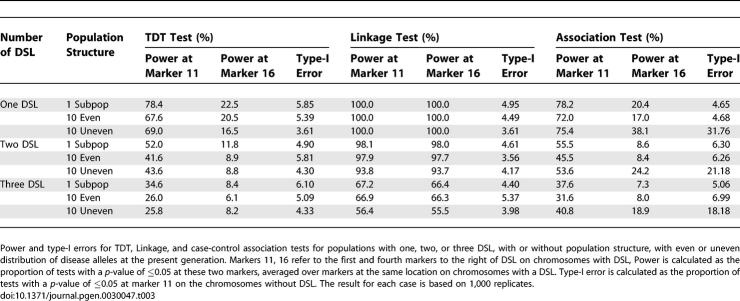
Power and Type-I Errors of the Gene-Mapping Methods Used in Example 1

It is evident that all methods have good power for cases with one DSL and no population structure, but the power decreases with an increasing number of DSL. Among three gene-mapping methods, the Linkage method performs best. It not only detects 100% of all DSL in the one-DSL cases, but also maintains good power when the number of DSL increases, and at markers further away from the DSL. For example, the power of the Linkage method is almost unchanged when the number of DSL increases from one to two, while the power of TDT and association tests decreases approximately 25%. In the meantime, the Linkage method has the best power at marker 16 compared with the TDT and association tests for cases with one or two DSL. The clear advantages of the Linkage method may reflect the fact that linkage is maintained over longer physical distances than association, but may also be due to our choice of single-locus TDT and association tests. In this example, we assume a moderately dense marker map with marker 11 at about 25 kbp from the DSL. This density does not provide a high level of linkage disequilibrium that is needed by the TDT method to outperform the Linkage method.

The TDT and Linkage methods keep reasonable type-I errors in all scenarios, including cases with population structure. The type-I errors for association tests are close to the nominal level for cases without population structure and cases with population structure but equal disease allele frequencies among subpopulations (the even case). For cases with uneven disease allele frequencies, the association method yields highly inflated type-I errors. This is a well-known defect of association studies [45–47] and the fact that equal disease allele frequencies among subpopulations restore correct type-I error confirms the results that the effects of population structure can be eliminated by carefully matching cases and controls according to self-reported ancestry and geographical origin [48,49].

If spurious associations are well controlled, case-control association tests are slightly more powerful than TDT method. Note that this comparison is based on the same sample size (800) but different sample types (200 affected sibpair families versus 800 unrelated individuals).

### Example 2: Mapping a Quantitative Trait Using Small or Large Pedigrees

Table 4 lists the powers and type-I errors of variance components [20] and variance regression [21] methods in mapping the quantitative trait from samples consisting of sibpair families or large pedigrees, based on 3,000 replicates.

**Table 4:**
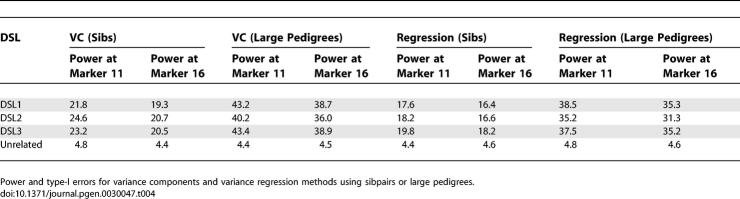
Power and Type-I Error for Example 2

The variance components method is more powerful than the variance regression method for both types of samples. Both methods have much better power in detecting DSL associated with the quantitative trait using samples of large pedigrees than samples of sibpair families. This indicates that large pedigrees provide greater power per genotyped individual than small pedigrees [50]. Further analyses can be performed to determine the answers to other questions such as how many large pedigrees are needed to achieve the same power as studies using 200 sibpair families.

Both methods have similar power in detecting all three DSL, although there is some evidence that DSL 2 is harder to map than others. This reflects the fact that three DSL have the same present disease allele frequencies and disease alleles at these DSL have the same impact on the quantitative trait. If we compare the power in detecting DSL from markers further away than marker 11, the power at marker 16 of both methods is similarly lower than at marker 11. This indicates that these two methods respond similarly to the reduction of linkage with increasing physical distance along the chromosome.

Finally, both methods have type-I errors close to a nominal level of 0.05. It would be interesting to distort the distribution of the quantitative trait and evaluate the impact of non-normality (or transformation methods) on the power of both methods because the variance components method is more sensitive to non-normality than is the variance regression method [21,51].

### Example 3: Age of Onset of a Hypothetical Cancer Caused by Three Interacting DSL

We simulate 2,000 replicates of the population and simulate age of onset and year of birth for each individual. For a typical population, the population prevalence of the five groups of individuals, as in Equation 7, are 0.94%, 8.28%, 0.46%, 4.02%, and 86.28%, respectively, and the average age of onset, conditioning on affection status before age 75, are 27.0, 40.9, 36.7, 39.7, and 45.7 y, respectively. The percentage of unaffected individuals (i.e., those with age of onset >75) in these groups are 6.3%, 43.0%, 26.9%, 35.8%, and 69.8%.

The first type of samples consists of cases with 400 affected individuals and 400 unaffected individuals older than 50 y. The population haplotype frequencies of the five groups of individuals (Equation 7), among cases and controls, are (3.8% and 0.1%), (15.2% and 6.1%), (1.2% and 0.2%), (8.6% and 2.5%), and (71.1% and 91.1%). We apply logistic regression to regress affection status on DSL and an unrelated marker, using Model 1: Affection ∼Marker + DSL1 + DSL2 + DSL3, Model 2: Affection ∼ Marker + DSL1*DSL2*DSL3, and Model 3: Affection ∼ Marker + DSL1:DSL2 + DSL1:DSL3 + DSL1:DSL2:DSL3, which represent models with independent DSL, all possible interactions, and true interaction items, respectively. We use notations from R in which “+” stands for additivity, “:” for interaction, and “*” for all interaction terms. We then apply Cox proportional hazards model using similar models but with the affection status replaced by the survival function estimated from age of onset and affection status. Here, we consider the onset of disease as terminal event and use age of onset as the survival time for affected individuals, and use age as right-censored survival time for unaffected individuals.

For the second type of samples, the cases are 800 affected individuals with early age of onset (<40 y), and the controls are 800 affected individuals with an age of onset ≥40 y. The population haplotype frequencies of the five groups of individuals (Equation 7), in the case and control groups, respectively, are 6.0% and 3.2%; 16.4% and 14.9%; 1.4% and 1.2%; 10.1% and 8.2%; and 66.0% and 72.6%. We apply logistic regression with the same set of models as the first design.

The power and type-I error, estimated as the percentage of replicates that yield a *p*-value of ≤0.05 for each dependent item, are presented in Table 5. We first noticed that the type-I errors of all the models, estimated using an unrelated marker, are close to the nominal level 0.05, which is reassuring. Although none of the disease alleles contributes alone to the onset of the disease, the marginal effects can be detected using models that assume independent DSL (Model 1). These marginal effects are stronger than interacting effects (Model 3), although this may be because there are three genotype states at each DSL and nine states at two interacting DSL. If we regress on all possible interacting items (Model 2), both marginal and interaction effects are reduced. This calls for the use of variable selection procedures to identify significant effects.

**Table 5:**
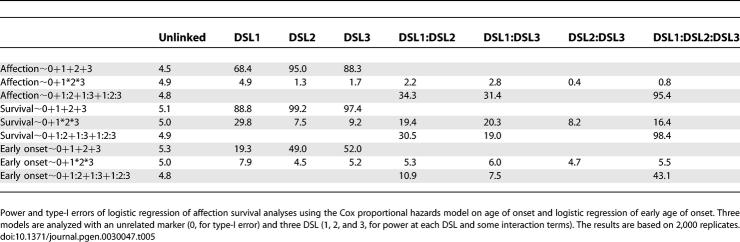
Power and Type-I Error for Example 3

The use of age of onset (and age for the unaffected individuals) and survival analyses generally increases the power of detecting these DSL. This may be because survival analyses make use of more information than does logistic regression. The analyses for early age of onset show similar patterns of significance. Because the allele frequency differences are smaller for this design, the power of all models is lower, even with a doubled sample size.

## Discussion

We propose a forward-time simulation framework to simulate the evolution of complex human diseases and generate large virtual populations from which various types of samples can be drawn and analyzed. For example, we can map genes associated with a disease using both family-based sibpair samples and individual-based case-control samples, or even a combination of the two, and compare the performance of different gene-mapping methods. Our approach provides maximum flexibility at the cost of computing time, although we believe that in a time when computing power is getting less and less expensive, the benefits of this approach can easily outweigh the need for faster but more specialized sample generators (e.g., SimPed [52] or SIMLA [53]) or write specialized simulation procedures. Another benefit of this approach is that it allows us to study the impact of past demographic and genetic features on the mapping of a disease.

There are some limitations to our methods. Although the backward trajectory simulation algorithm can handle mutation, selection, subpopulation structure, migration, population size changes, and simple gene–gene interaction, it cannot yet simulate trajectories of linked DSL. This is because this algorithm assumes independent allelic segregation for both the estimation of the fitness of genotype at a single DSL (Equation 6) and the prediction of allele frequency at the previous generation (Equation 4). If linked DSL are to be modeled, one can resort to the traditional forward-time simulation method [18], in which the disease alleles are manually brought to designed disease allele frequencies using strong positive selection and then allowed to evolve freely until the present generation. If the population size is large at the end of the mutant-introduction stage, the disease allele frequencies may not deviate too much from the designed frequencies at the present generation. It is possible to combine these two approaches and add free-evolving DSL to our simulation framework. This can be the topic of further study.

The assumption that all mutants are derived from a single ancestral mutant can be problematic as well. For example, it is possible that a mutant has a high mutation rate and is subject to strong purifying selection. It may appear and disappear in the population more than once and has a nonnegligible impact on the evolutionary process. Because our backward trajectory simulation algorithm always obtains 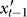 from *x_t_* = 0, our current method cannot model this process. A related problem is that we cannot simulate multiple mutants of different origins and multiple alleles at the same DSL.

Although our approach can control the allele frequencies of DSL at the present generation, it cannot control the allele frequencies of other markers, which are maintained by mutation and genetic drift. For a long enough simulation, many markers will become extinct or fixed (even when mutations can bring some of them back to life), and we have little control over the distribution of marker allele frequencies in the present generation. Simulations in our examples have very few (<0.01%) monomorphic markers due to rapid population expansion. These uninformative markers are ignored if they are tested for disease association. If the control of marker allele frequencies is important, one can start with a population with known marker allele frequencies and try to maintain the frequencies with rapid population growth or short evolution time. Resampling-based methods such as HAP-SAMPLE (F.A. Wright, H. Huang, X. Guan, K. Gamiel, C. Jeffries, et al., unpublished data) can also be used, although they assume no selection at the DSL.

The controlled random mating algorithm may not handle extremely high allele frequencies well, especially in the cases of multiple DSL. Because we accept families with disease alleles at any DSL that do not meet the allele frequency requirement, two problems may arise: (i) Disease alleles at other DSL will be accepted, even if their expected allele frequencies have been reached. Because high disease allele frequencies increase the likelihood of cosegregation of disease alleles, this problem is more pronounced in such cases. (ii) If the sum of all to-be-reached allele frequencies is larger than 1 (e.g., five DSL with 25% disease allele frequency), it is possible that some of the allele frequency requirements cannot be met when the offspring generation is filled.

Fortunately, the above problems rarely occur in reality. In extreme cases (Table 1) of five DSL at 50% each, only a few DSL of all replicates reach ≥51% allele frequency and there is no noticeable distortion of population statistics.

Proper use of this simulation framework also calls for careful selection of demographic and genetic models. Although hypothetical diseases can be simulated and studied, it might be more useful to simulate real diseases. In these cases, we should collect as much information as possible, including the demographic distribution of the disease alleles, the demographic history of the studied population, the possible number of DSL, past selective episodes, and the estimated ages of the mutants.

Despite heated debates [54,55], it is widely believed that the human population has continued to expand since the origin of modern humans 100,000–250,000 y ago [56,57], with an estimated initial effective population size between 700 [57] and 10,000 [56]. The expansion of modern humans was accompanied by migrations to other parts of the world and mostly happened separately in subpopulations. The “out of Africa” migration to Eurasia happened approximately 45,000–55,000 y (2,000–2,500 generations) ago, although some believe there were brief migration episodes before that [54,58]. This migration process can be modeled by a sequential colonization process in which a subpopulation only migrates to its adjacent subpopulations [59,60]. After subpopulations settle down, their sizes usually expand quickly and can largely be modeled by an exponential population growth model, with a few exceptions. For example, the Finland population is a good example of a recent and quickly expanded population, and the Saami population in northern Fenno-Scandinavia can be used to study small populations of constant size [29].

Depending on the nature of simulations, we can choose different demographic models to simulate the demographies of different populations. If little is known about the demographic feature of a disease, we can assume a general model, which is largely the one we used in our examples. The key parameters are an initial population size of approximately 5,000, final population size ≥5 × 10^5^_,_ ≥4,000 burn-in generations, and ≥4,000 evolution time, with the population split occurring approximately 2,000 generations ago if population structure is to be modeled.

A genetic model for the DSL depends on the current disease allele frequency and the demographic model used. From a simulation point of view, although there are trajectories for any demographic and genetic settings, the trajectory simulation algorithm will fail to generate an unlikely trajectory after 1,000 attempts. For example, it is difficult to simulate trajectories with strong purifying selection and high present disease allele frequency without the help of demographic features such as a bottleneck. This problem is more severe for multiple interacting DSL because their fitnesses are frequency dependent and can oscillate between purifying or advantageous. As a matter of fact, all three DSL in Example 3 can be under purifying selection, and it is unlikely to simulate higher disease allele frequencies (e.g., 50%, 40%, or 40%) under the fitness model presented in Table 2.

Although known mutants for various diseases are generally young (less than 170 generations for mutation C282Y on human HFE allele [61,62] and many others [63]), their abundance may be an artifact of ascertainment bias because younger mutants are easier to map. Moreover, special hypotheses, such as small population size caused by population structure and the existence of population bottleneck, are often needed to explain these young alleles. Using our simulation framework, the high allele frequency can be simulated by population expansion after a bottleneck [56], by positive selection pressure of constant intensity [62,64], or other mechanisms such as antagonistic pleiotropy (alleles have a selective advantage before reproduction age, followed by selective disadvantage in later life [65]), changing selection pressure due to environmental or social changes [11], or a heterozygote advantage. Although hitchhiking (a disease allele tightly linked to another locus that is under positive selection) is a possibility, our simulation framework cannot simulate it because it involves linked DSL. Note that demographic features are less important for younger mutants (e.g., under strong positive selection) than older ones.

The power, and perhaps the weakness, of our method lies in the modeling of the evolutionary history of complex human diseases. If we assume that DSL are neutral so the evolutionary history has little impact on the mapping of the disease, simulating the whole evolutionary history may be cumbersome and unnecessary. However, we believe that past demographic and genetic features have a strong impact on the genetic composition of the present human population, and the modeling of the evolutionary history of complex human diseases would help develop more powerful gene-mapping methods. For example, one can study the impact of age of the population admixture on the power of admixture mapping [66], or the impact of advantageous selection, which can cause transmission distortion [67], on the gene-mapping methods used in Example 1.

## Supporting Information

Dataset S1 Forward-Time Simulations of Human Populations with Complex Diseases (152 KB DOC)

## Abbreviations

DSL: disease susceptibility loci
LD: linkage disequilibrium
MRCA: most recent common ancestor
