# Supplementary material for: Forward-Time Simulations of Human Populations with Complex Diseases
Source: PLoS Genet. 2007 Mar 23;3(3):e47. doi: 10.1371/journal.pgen.0030047 (PMC1829403; doi:10.1371/journal.pgen.0030047)
Supplement: Supplementary file 1 [file pgen.0030047.sd001.doc]

# Supplementary data: Forward-time Simulations of Human Populations with Complex Diseases

Given the disease allele frequencies and the penetrance model, it is possible to estimate some population properties theoretically. These estimates will be compared with simulated populations to validate our simulations. Many of the results are multi-locus extensions of the two-loci cases discussed in [1].

We assume that there are $L$ unlinked binary DSL and the disease allele frequency at locus $i$ is $f_{i}$ , $i=1,\text{.}\text{.}\text{.}, L$ . Let the genotype at a DSL be $g_{i}=g_{i}^{1}g_{i}^{2}$ , $i=1,\text{.}\text{.}\text{.}, L$ , where $g_{i}^{j}$ can be $N=1$ or $S=2$ . The frequency of a particular genotype is $F\left( g \right)=F\left( g_{i}, i=1,\text{.}\text{.}\text{.}, L \right)$ , which, assuming no interaction between DSL and homologous genotypes, is equal to

$$\begin{aligned} F(g)=\underset{}{\overset{i=1}{L}}\prod F_{i}\left( g_{i} \right)=\underset{}{\overset{i=1}{5}}\prod\left[ f_{i}^{g_{i}^{1}-1}\left( 1-f_{i} \right)^{2-g_{i}^{1}} \right] \left[ f_{i}^{g_{i}2-1}\left( 1-f_{i} \right)^{2-g_{1}^{2}} \right] \end{aligned}$$

where $F_{i}\left( g_{i} \right)$ is the genotype frequency at DSL $i$ .

We use a heterogeneity penetrance model based on an additive single-locus penetrance model. Given single locus penetrance parameter $\delta_{i}$ , the penetrance of an individual is

$$P(g)=1-\underset{i=1}{\overset{L}{\prod}}[1-P_{i}(g_{i})]$$

where $P_{i}\left( g_{i} \right)$ is 0, ${\delta_{i}}/2$ , or $\delta_{i}$ for genotype $\text{NN}$ , $\text{NS}(\text{SN})$ , or $\text{SS}$ .

Overall disease prevalence ( $K$ )

Let $G$ be all possible genotypes at the DSL ( $4^{L}$ distinct types). The population prevalence $K$ is

$$K=\underset{g\in G}{\sum}F\left( g \right) P\left( g \right)=\underset{g}{\sum}$$

which can be written as

$K=1-\underset{i=1}{\overset{L}{\prod}}\left( 1-K_{i} \right)$ (1)

where $K_{i}$ is the prevalence summands defined as

$$K_{i}=\underset{g_{i}\in G_{i}}{\sum}F_{i}\left( g_{i} \right) P_{i}\left( g_{i} \right)\text{.}$$

To prove (1), we first prove $F\left( g \right) P_{i}\left( g \right)$ and then simplify $\left( 1-p_{j}\left( g \right) \right)$ . Note that under the additivity assumption, $K_{i}$ can be simply expressed as $K_{i}=2\delta_{i}f_{i}$ .

Allele and genotype frequency in case and control groups ( $P_{\text{11}}, P_{\text{12}}, P_{\text{22}}$ , $f_{i}^{'}$ )

For any genotype $g$ ,

$$\begin{aligned} \text{Pr}\left( g | \text{affected} \right)=\frac{\text{Pr}\left( \text{affected}, g \right)}{\text{Pr}\left( \text{affected} \right)}=\frac{P\left( g \right) F\left( g \right)}{K} \\ \text{Pr}\left( g | \text{unaffected} \right)=\frac{\left( 1-P\left( g \right) \right) F\left( g \right)}{1-K}\text{.} \end{aligned}$$

Given $\text{Pr}\left( g | \text{affected} \right)$ , we can easily calculate the genotype frequencies at one DSL. For example,

$$\begin{aligned} \text{Pr}\left( g_{1} | \text{affected} \right)=\underset{g_{i}, i=2,\text{.}\text{.}\text{.}, L}{\sum}\text{Pr}\left( g_{i}, i=1,\text{.}\text{.}, L | \text{affected} \right)=\underset{g_{2}g_{3}g_{4}g_{5}}{\sum}\frac{P\left( g \right) F\left( g \right)}{K}\text{.} \end{aligned}$$

The sample disease allele frequency can then be written as

$$f_{i}^{'}=\frac{1}{2}\text{Pr}\left( \text{SN}\text{ or }\text{NS} | \text{affected} \right)+\text{Pr}\left( \text{SS} | \text{affected} \right)\text{.}$$

We report $P_{\text{11}}=\text{Pr}\left( \text{NN} | \text{affected} \right)$ , $P_{\text{12}}=\text{Pr}\left( \text{NS}\text{ or }\text{SN} | \text{affected} \right)$ , $P_{\text{22}}=\text{Pr}\left( \text{SS} | \text{affected} \right)$ , and $f_{i}^{'}$ .

Sibling recurrence risk ( $K_{s}$ and $\lambda_{S}$ )

Let $X_{P}$ , $X_{S}$ be the affection status of a proband and his or her sibling, respectively (1 for affected and 0 for unaffected). Let $K_{S}=E\left( X_{S} | X_{P}=1 \right)=P_{r}\left( X_{S}=1 | X_{P}=1 \right)$ be the recurrence risk for a sibling of an affected proband and $\lambda_{S}={K_{S}}/K$ be the risk ratio for the sibling of an affected individual compared with population prevalence. Therefore,

$$K\times K_{S}=E\left( X_{P} \right) E\left( X_{S} | X_{P}=1 \right)=E\left( X_{S}X_{P} \right),$$

where $K=E\left( X_{P} \right)$ is the population prevalence of the disease.

Let $F\left( g \right) P_{i}\left( g \right)$ and $K_{\text{Si}}=E\left( X_{\text{Si}} | X_{\text{Pi}}=1 \right)$ be $K_{S}$ restricted to DSL $i$ (using penetrance at DSL $i$ as individual penetrance). Let $g_{S}$ be the genotype of the sibling of the proband and $g_{p}$ be the genotype of the proband,

$$\tau\left( g_{s} | g_{p} \right)=\text{Pr}\left( g_{s} | g_{p} \right)$$

Conditioning on $g_{p}$ , we have

$$\begin{aligned} K\times K_{S}=\underset{g_{p}\in G}{\sum}\text{Pr}\left( X_{S}=1, X_{P}=1 | g_{p} \right) F\left( g_{p} \right)=\underset{g_{p}\in G}{\sum}F\left( g_{p} \right) P\left( g_{p} \right) \underset{g_{S}\in G}{\sum}\tau\left( g_{s} | g_{p} \right) P\left( g_{S} \right)\text{.} \\ \end{aligned}$$

By defining single locus measures such as $\tau_{i}\left( g_{\text{si}} | g_{\text{pi}} \right)=\text{Pr}\left( g_{\text{si}} | g_{\text{pi}} \right)$ , we can break into single locus measures, and we conclude that

$$K\times K_{s}=1-2\underset{i=1}{\overset{L}{\prod}}\left( 1-K_{i} \right)+\underset{i=1}{\overset{L}{\prod}}\left( 1-2K_{i}+K_{i}K_{\text{Si}} \right),$$

where $K_{\text{Si}}=E\left( X_{\text{Si}} | X_{\text{Pi}}=1 \right)$ is $K_{S}$ restricted to DSL $i$ (using penetrance at DSL $i$ as individual penetrance). This is an extension of expression (16) of Risch [1].

## Reference:

Risch N (1990) Linkage strategies for genetically complex traits. i. multilocus models. Am J Hum Genet 46:222--228.
